# Supplementary figures and images for: Degradation of benzo[a]pyrene by halophilic bacterial strain Staphylococcus haemoliticus strain 10SBZ1A
Source: PLoS One. 2021 Feb 25;16(2):e0247723. doi: 10.1371/journal.pone.0247723 (PMC7939701; doi:10.1371/journal.pone.0247723)

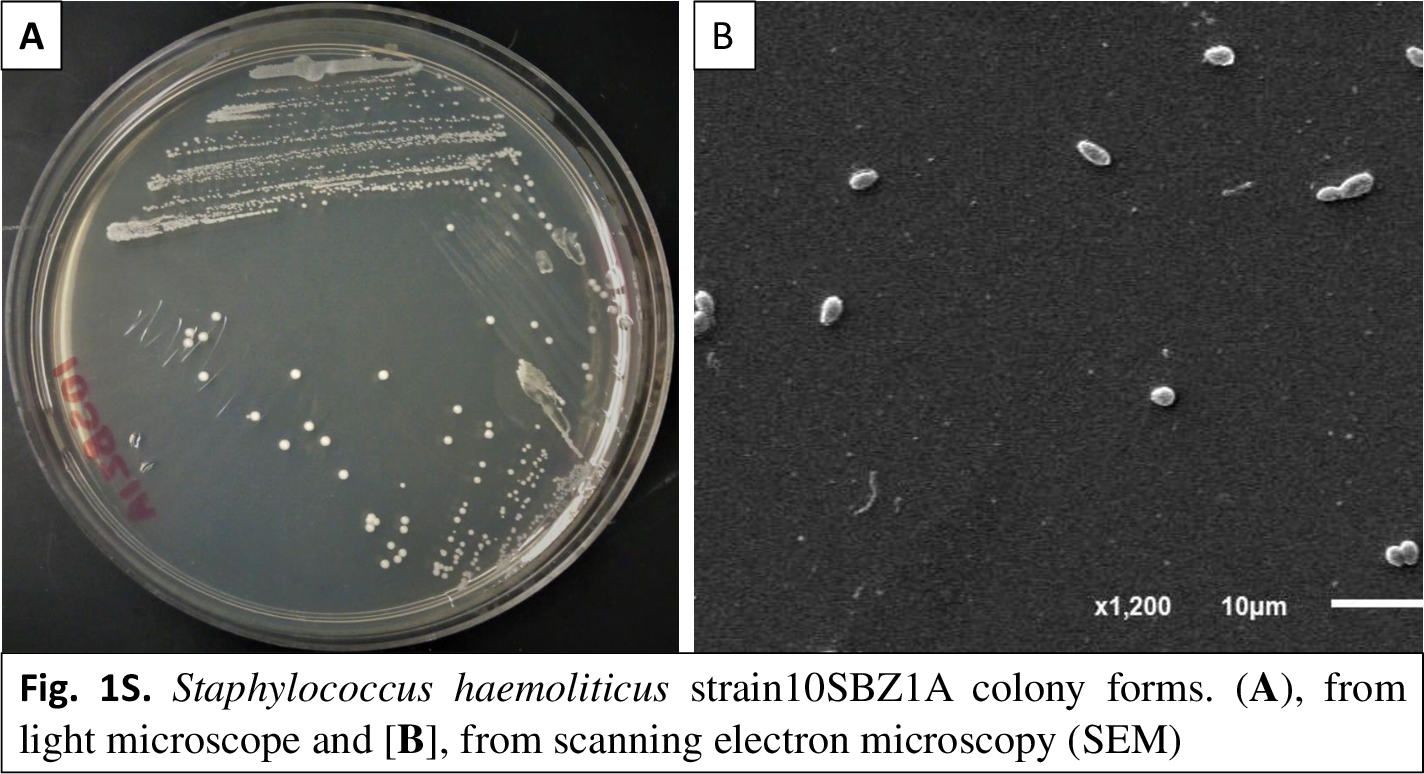

Supplement: S1 Fig — (A), from light microscope and [B], from scanning electron microscopy (SEM). (TIF) [file pone.0247723.s001.tif]

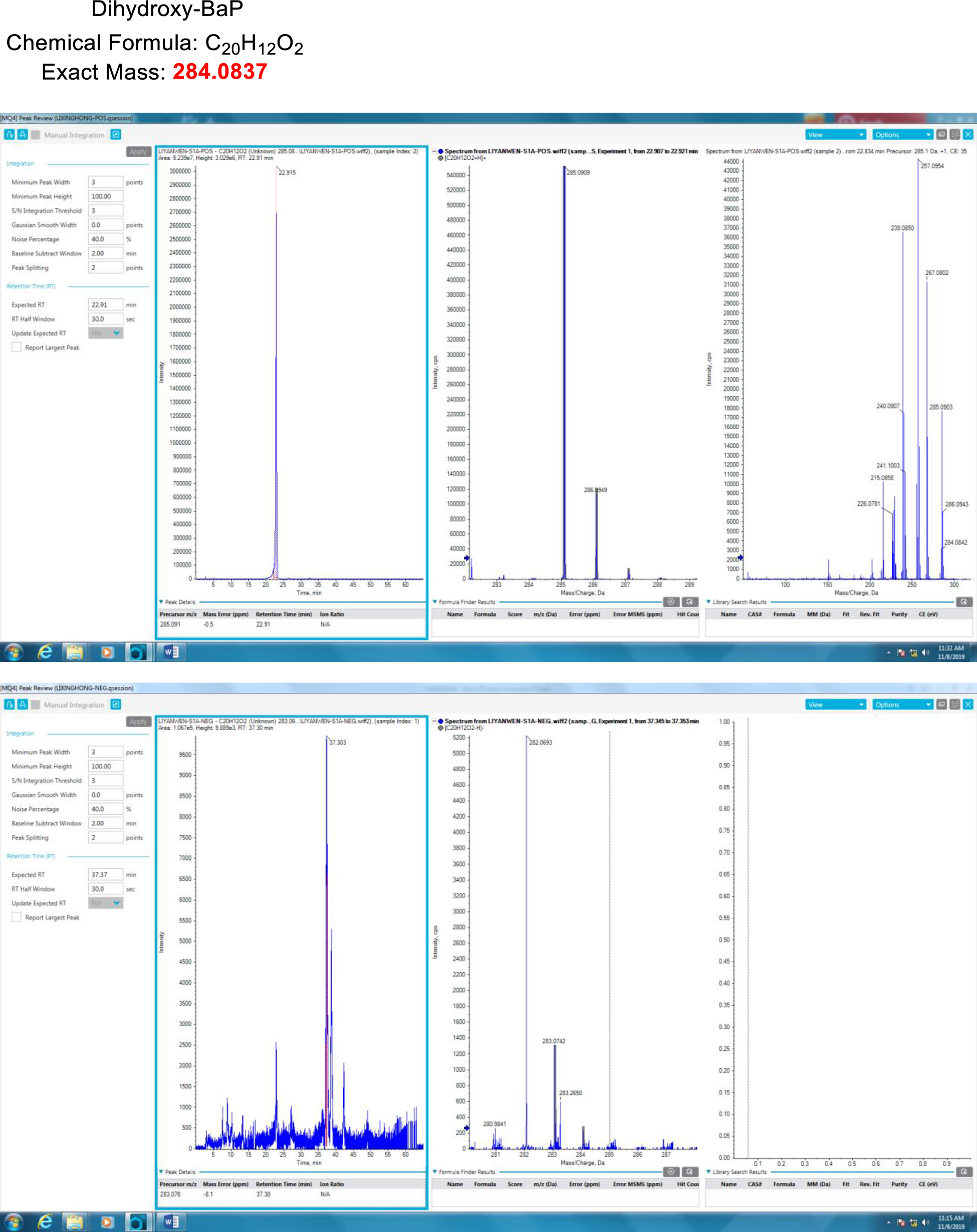

Supplement: S2 Fig — (ZIP) [file pone.0247723.s002.zip › PACE Corrected/Figure 2S Supplementary Material.tif]

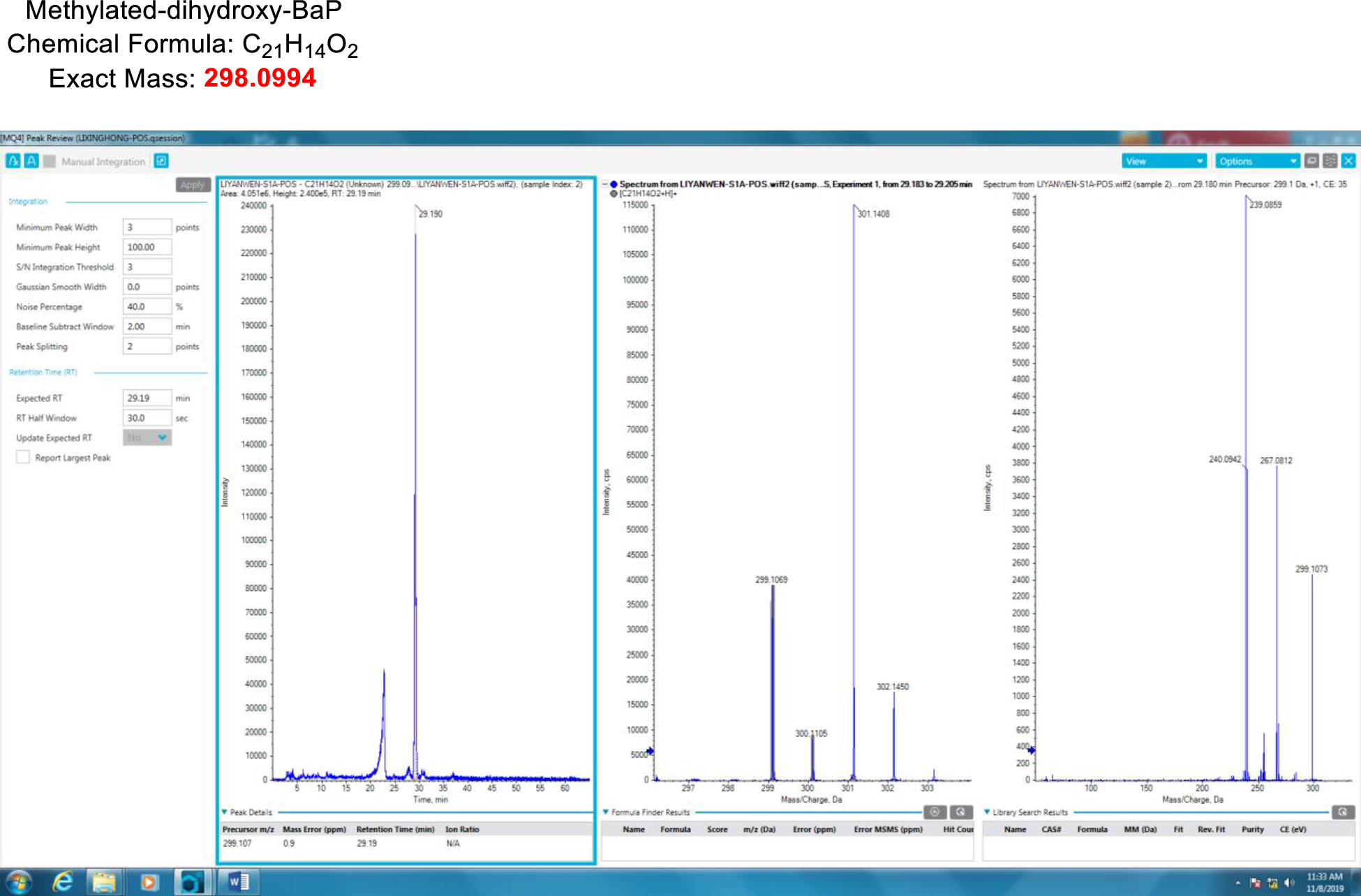

Supplement: S2 Fig — (ZIP) [file pone.0247723.s002.zip › PACE Corrected/Figure 2S Supplementary Material.tif]

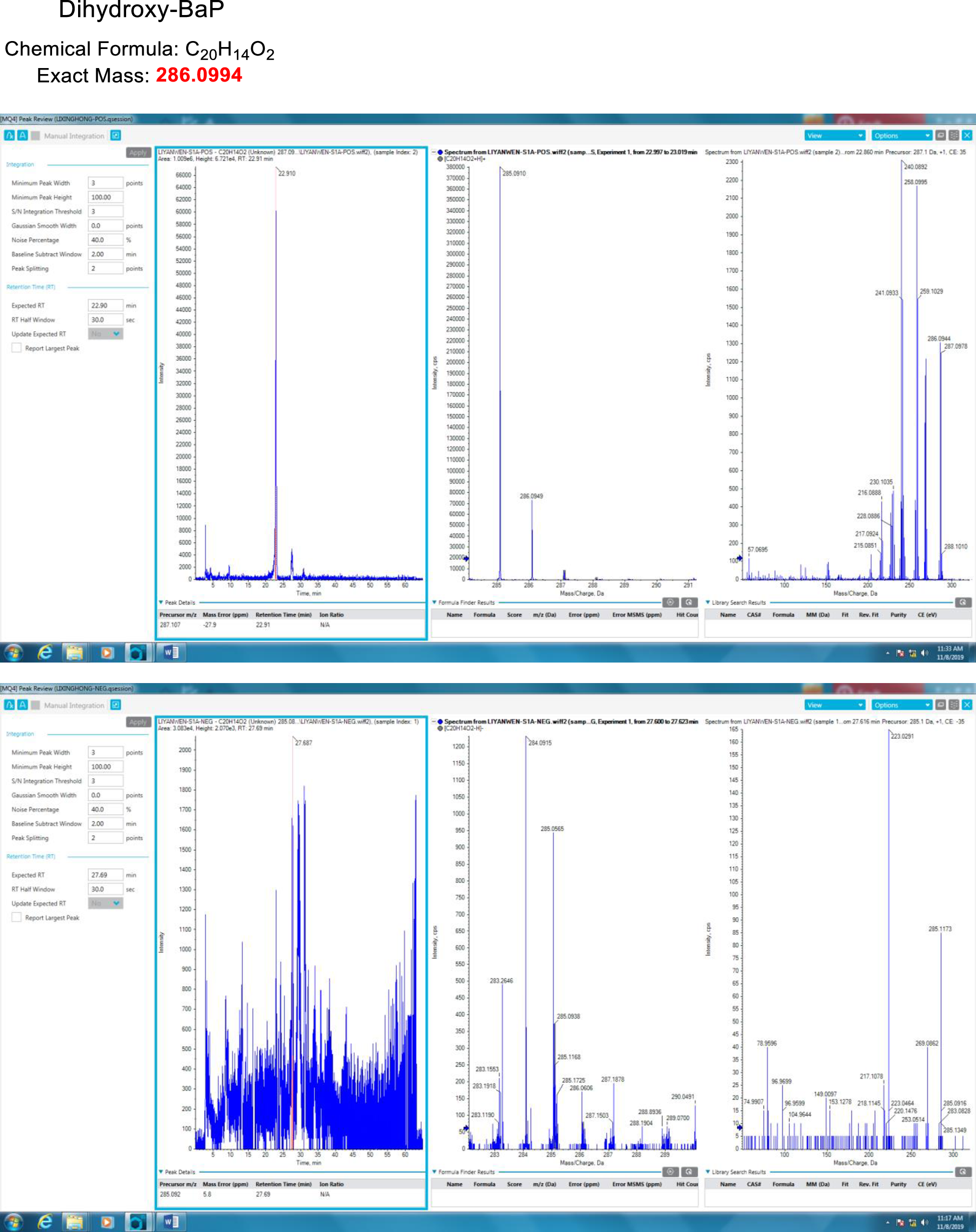

Supplement: S2 Fig — (ZIP) [file pone.0247723.s002.zip › PACE Corrected/Figure 2S Supplementary Material.tif]

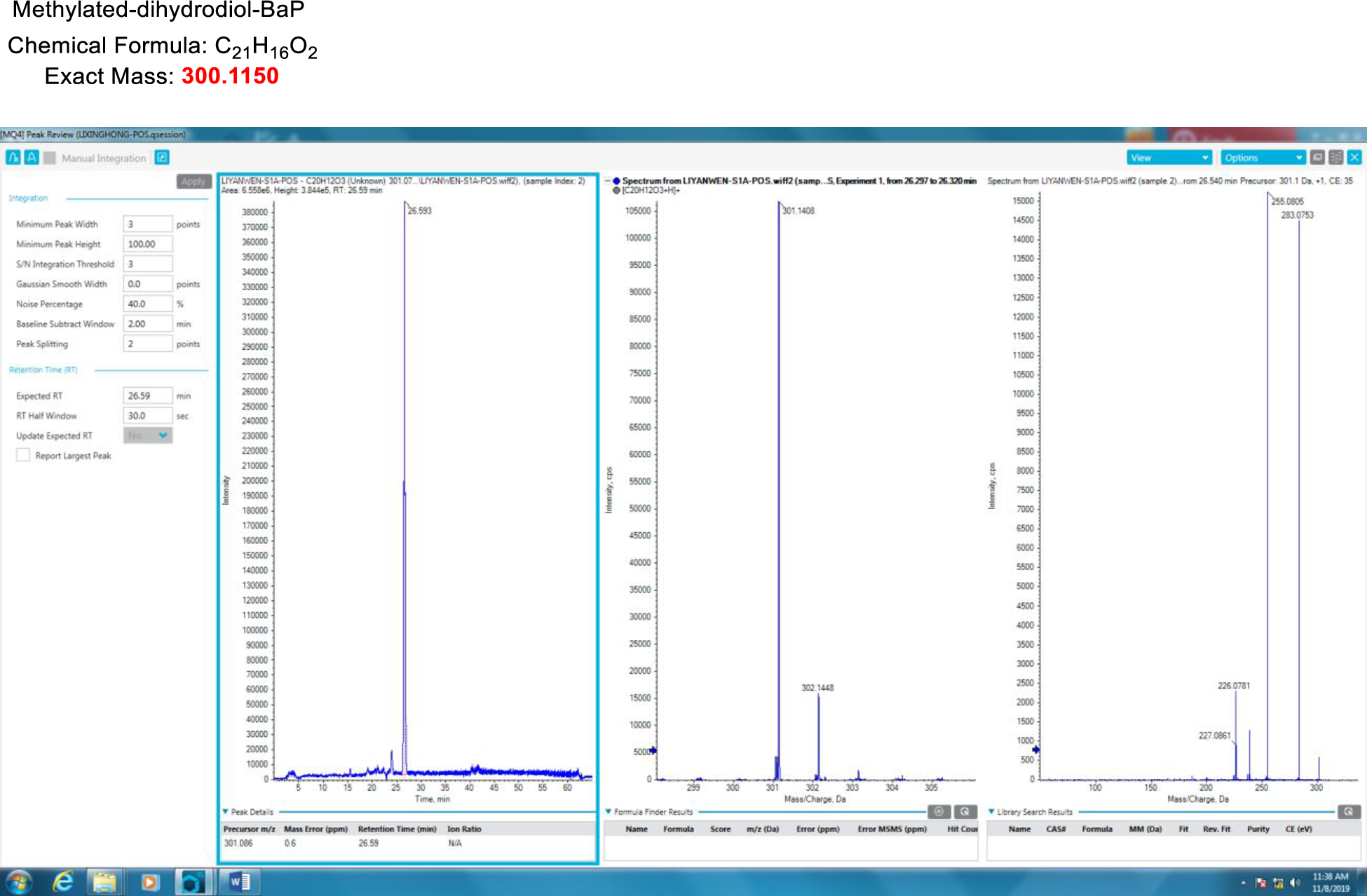

Supplement: S2 Fig — (ZIP) [file pone.0247723.s002.zip › PACE Corrected/Figure 2S Supplementary Material.tif]

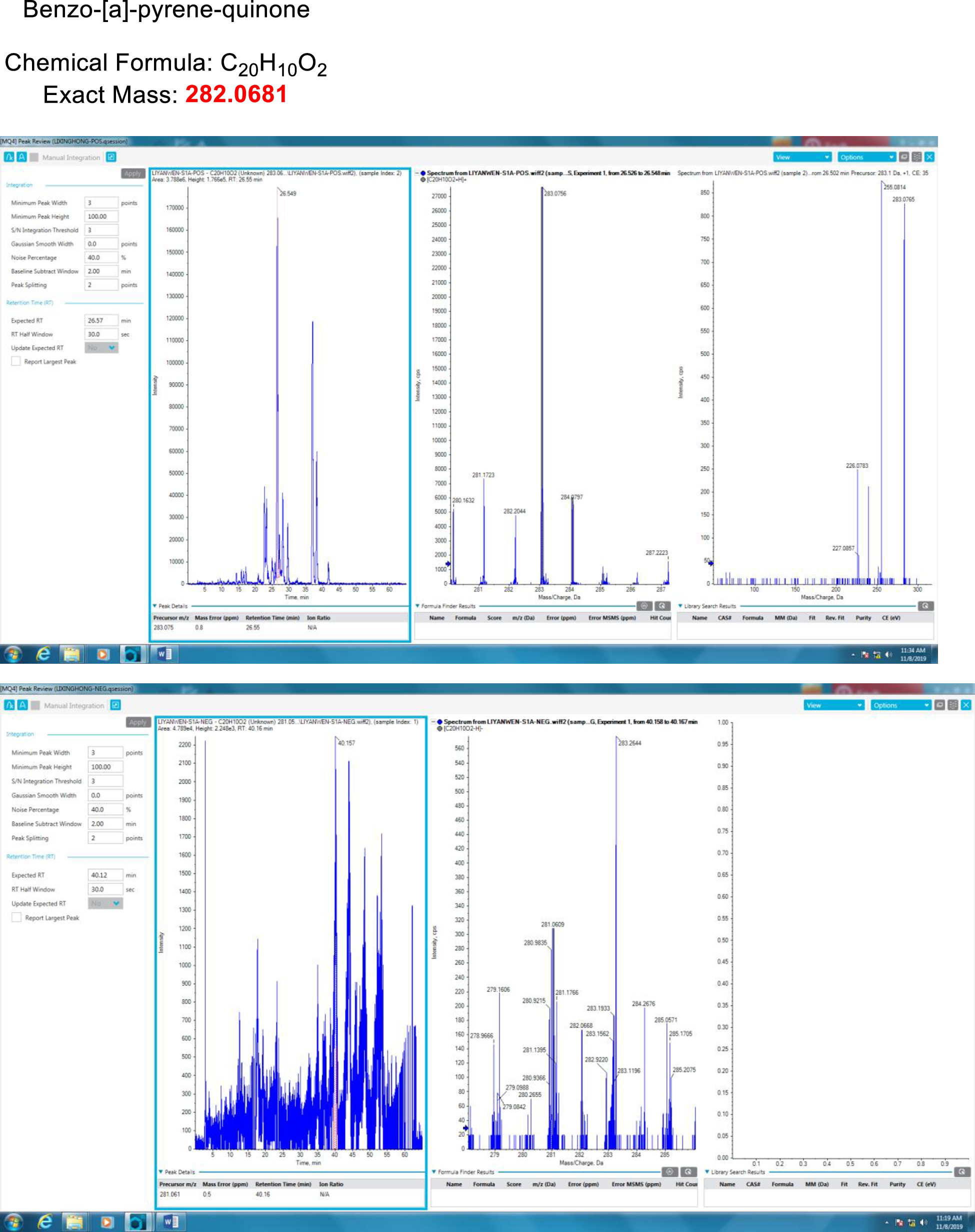

Supplement: S2 Fig — (ZIP) [file pone.0247723.s002.zip › PACE Corrected/Figure 2S Supplementary Material.tif]

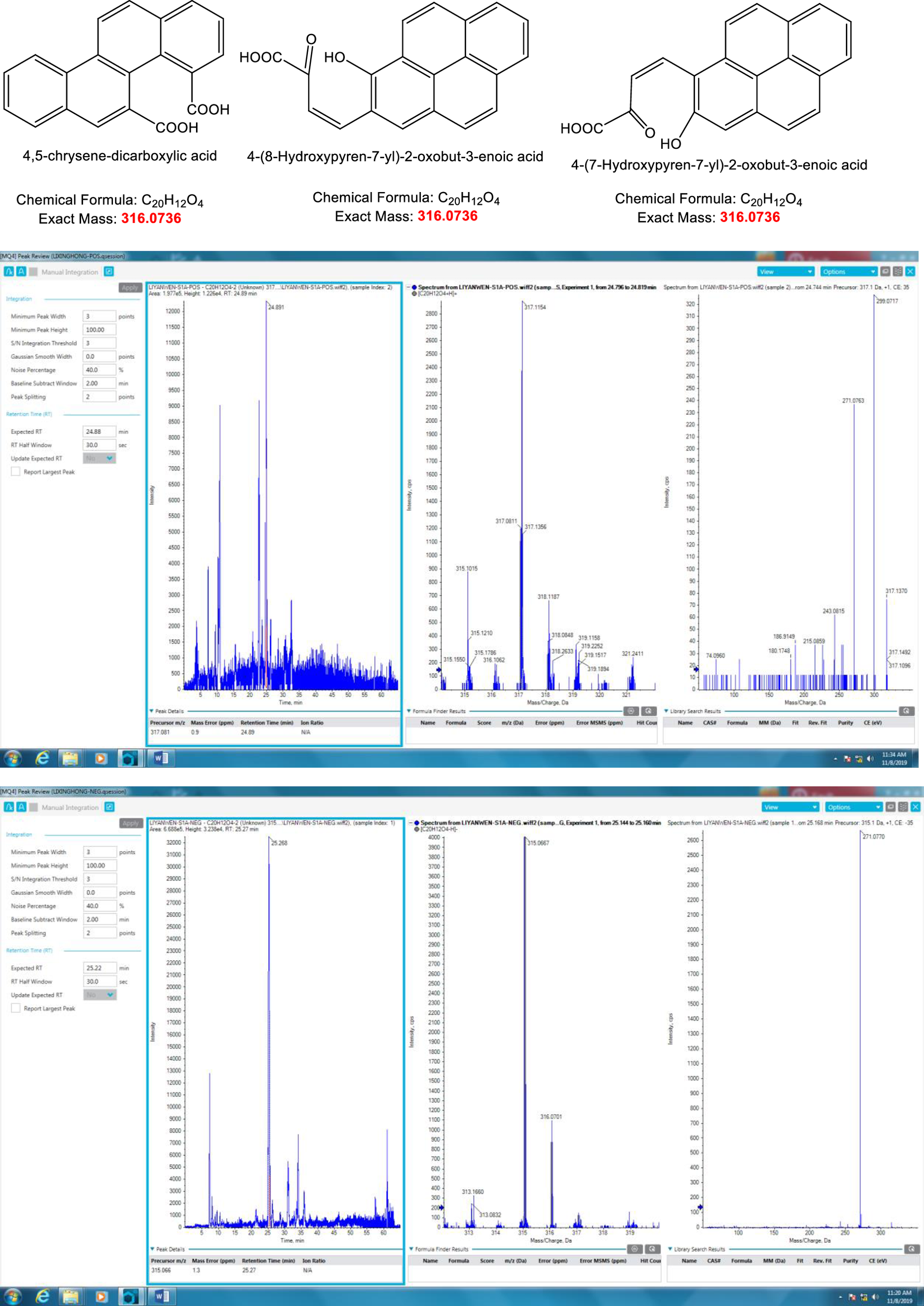

Supplement: S2 Fig — (ZIP) [file pone.0247723.s002.zip › PACE Corrected/Figure 2S Supplementary Material.tif]

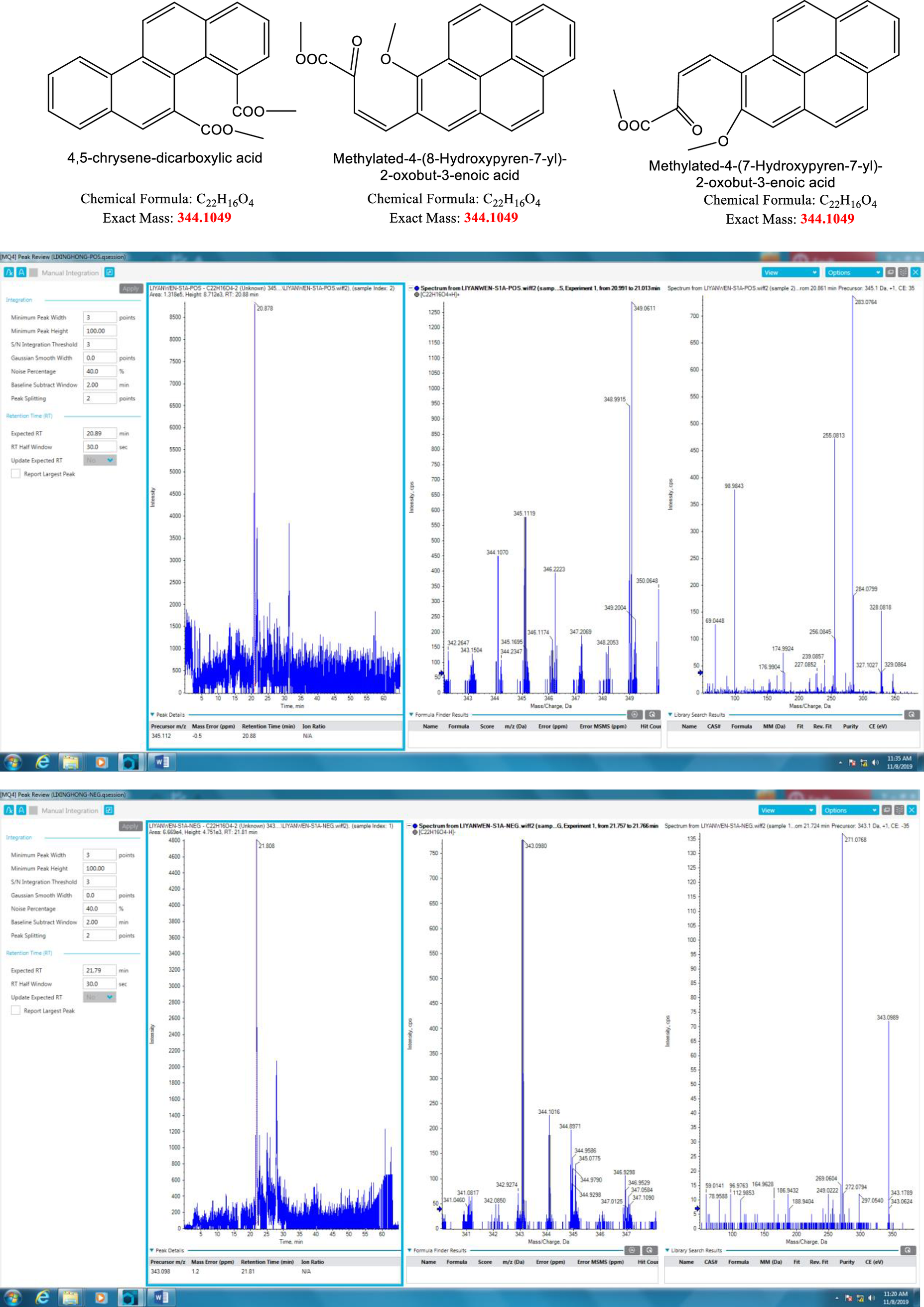

Supplement: S2 Fig — (ZIP) [file pone.0247723.s002.zip › PACE Corrected/Figure 2S Supplementary Material.tif]

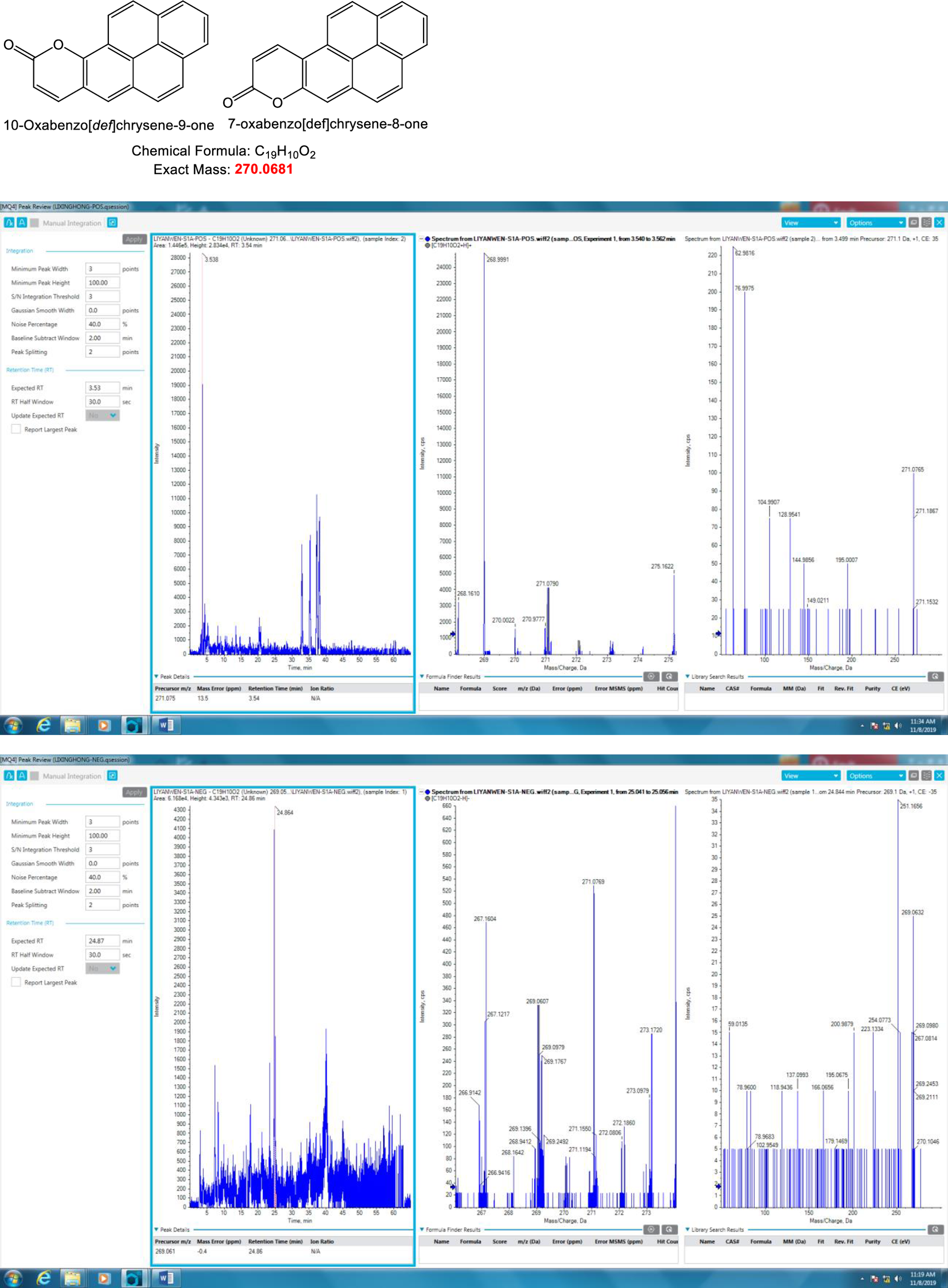

Supplement: S2 Fig — (ZIP) [file pone.0247723.s002.zip › PACE Corrected/Figure 2S Supplementary Material.tif]

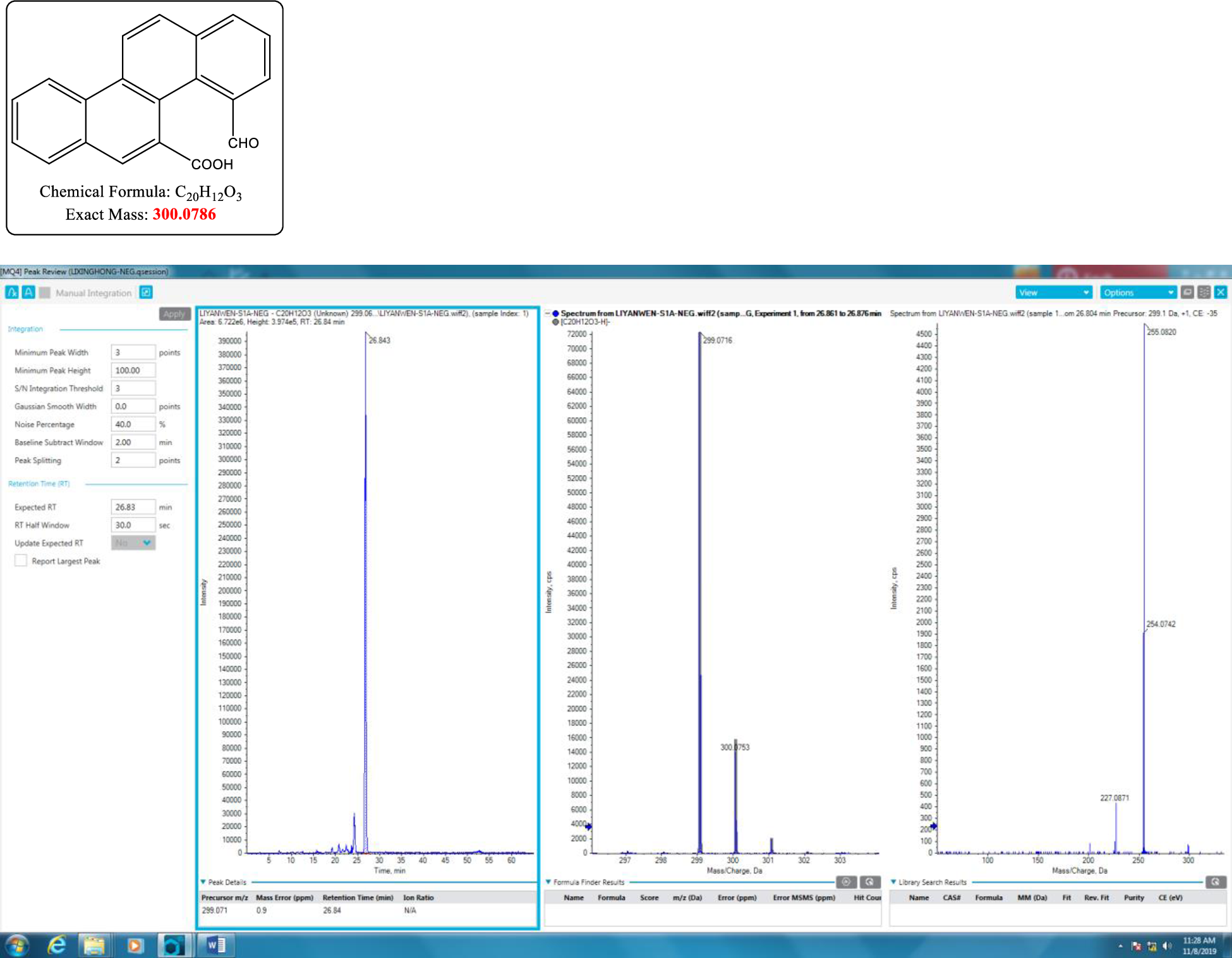

Supplement: S2 Fig — (ZIP) [file pone.0247723.s002.zip › PACE Corrected/Figure 2S Supplementary Material.tif]

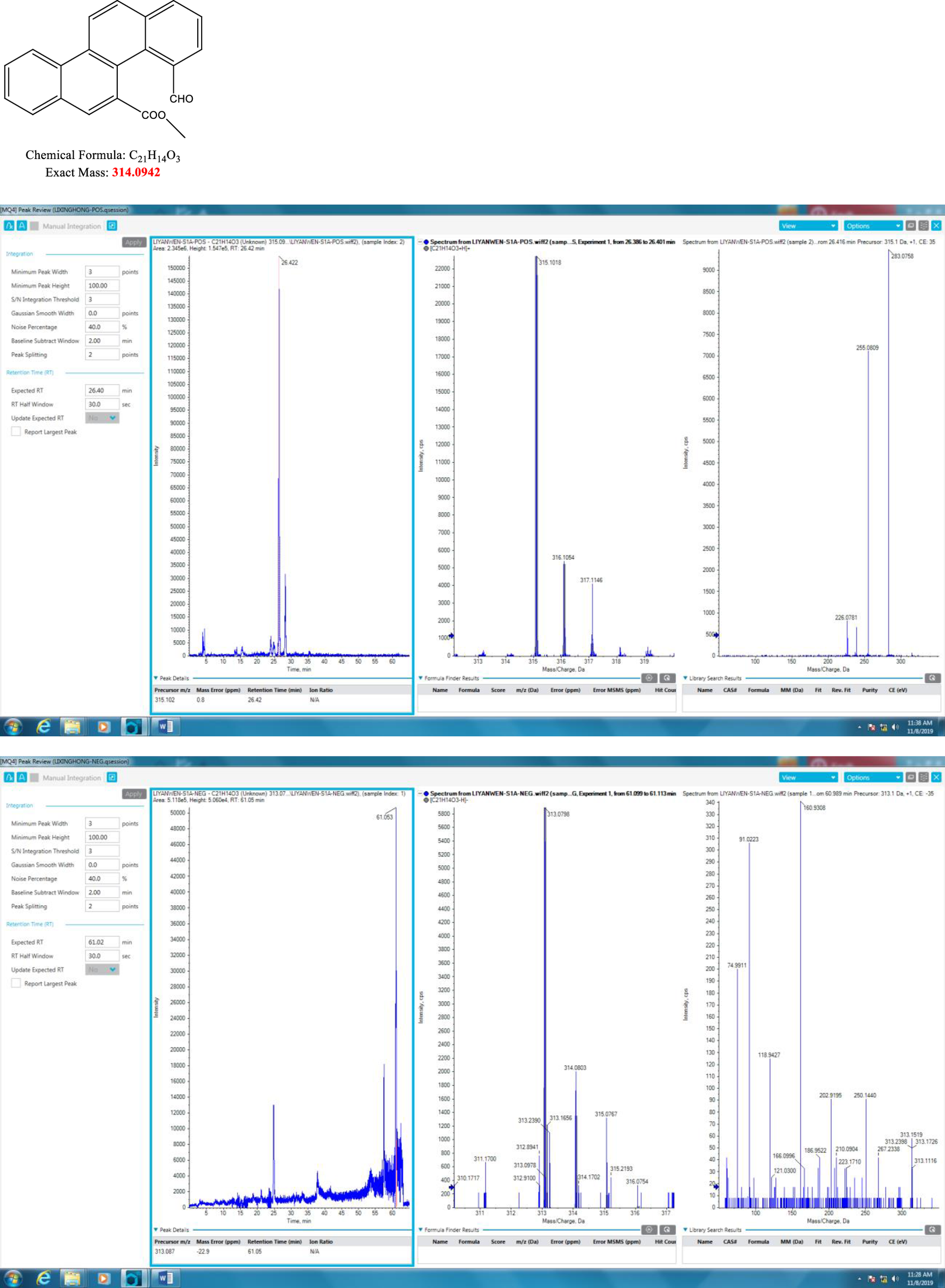

Supplement: S2 Fig — (ZIP) [file pone.0247723.s002.zip › PACE Corrected/Figure 2S Supplementary Material.tif]

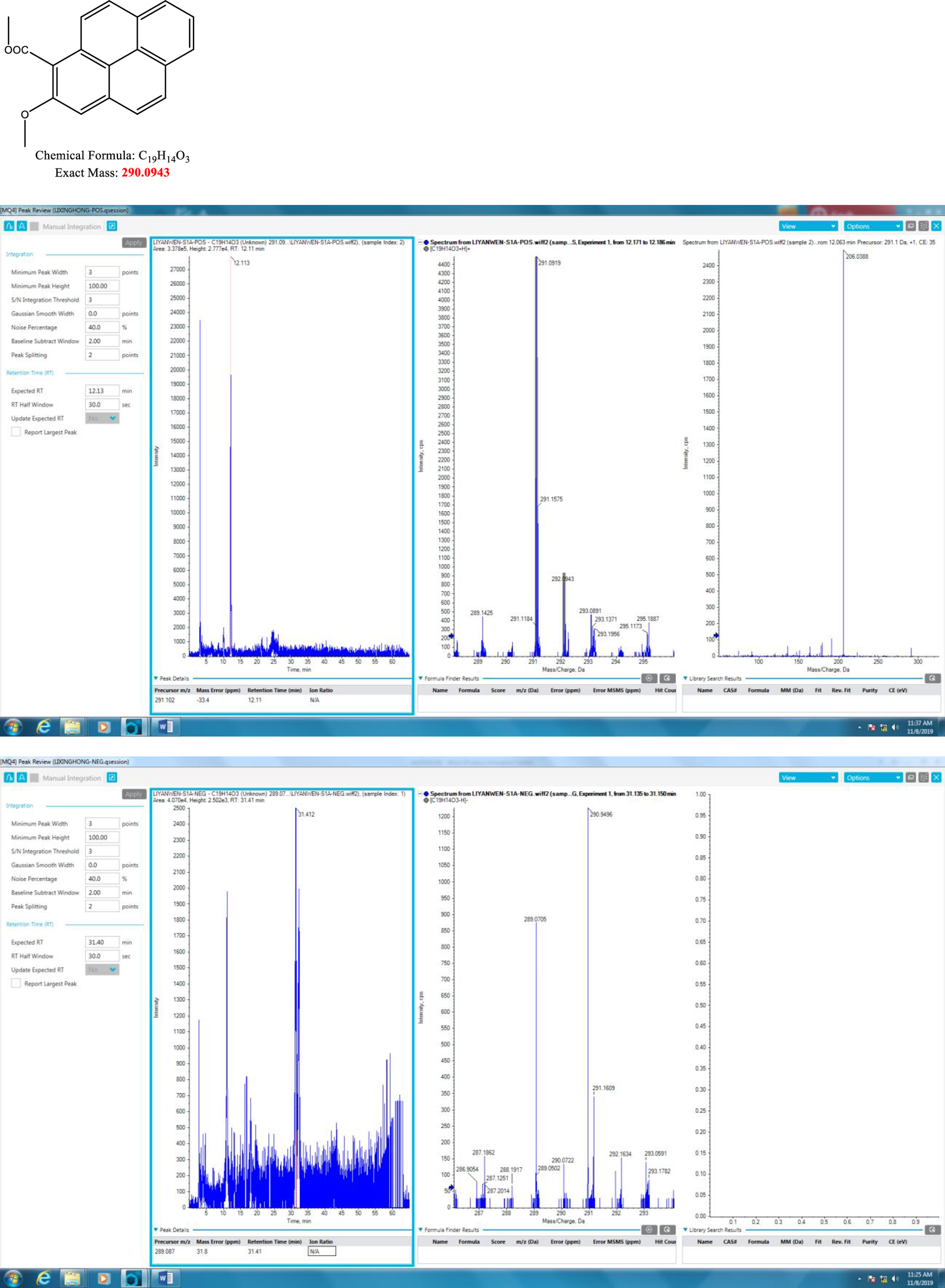

Supplement: S2 Fig — (ZIP) [file pone.0247723.s002.zip › PACE Corrected/Figure 2S Supplementary Material.tif]

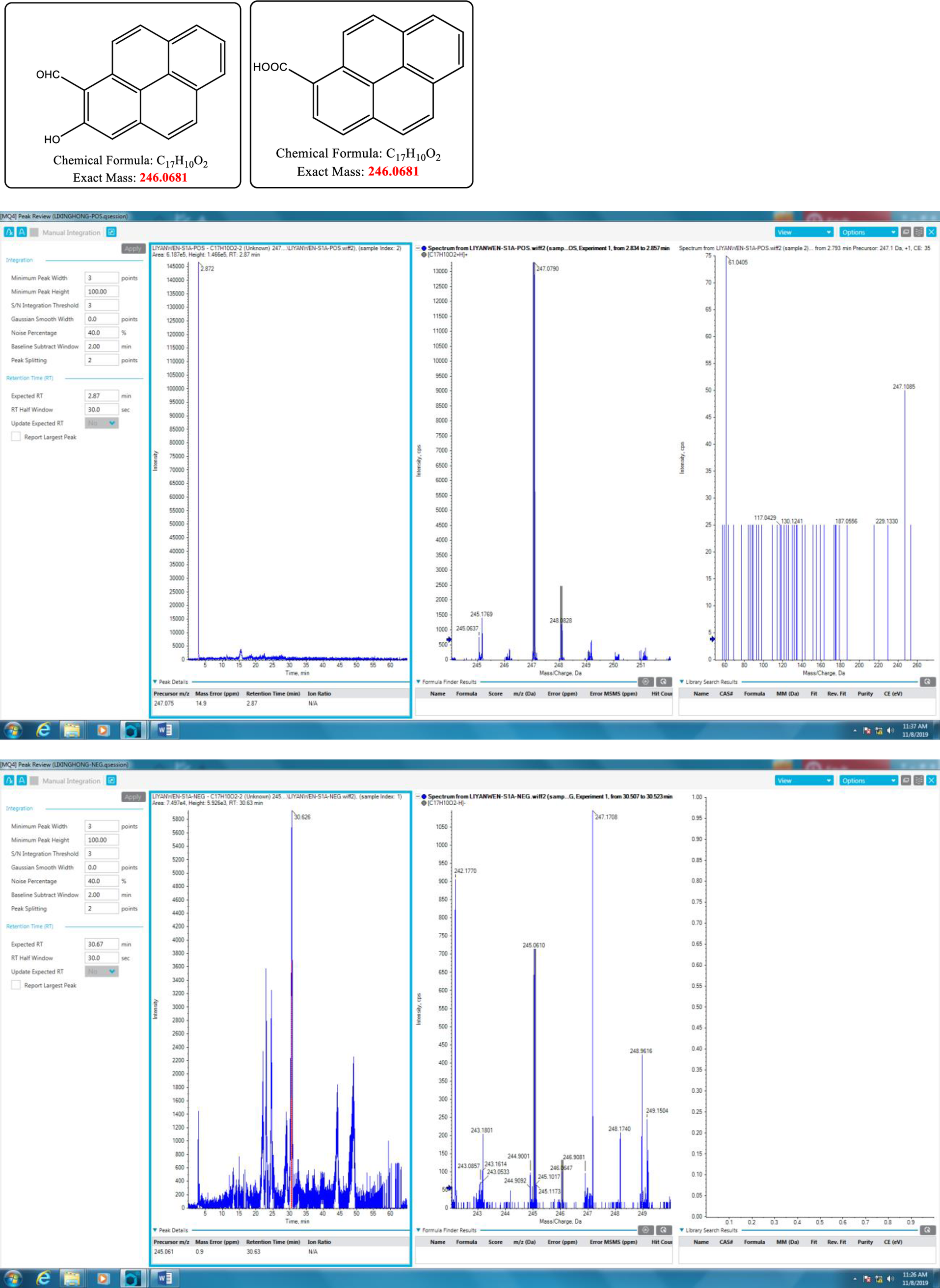

Supplement: S2 Fig — (ZIP) [file pone.0247723.s002.zip › PACE Corrected/Figure 2S Supplementary Material.tif]

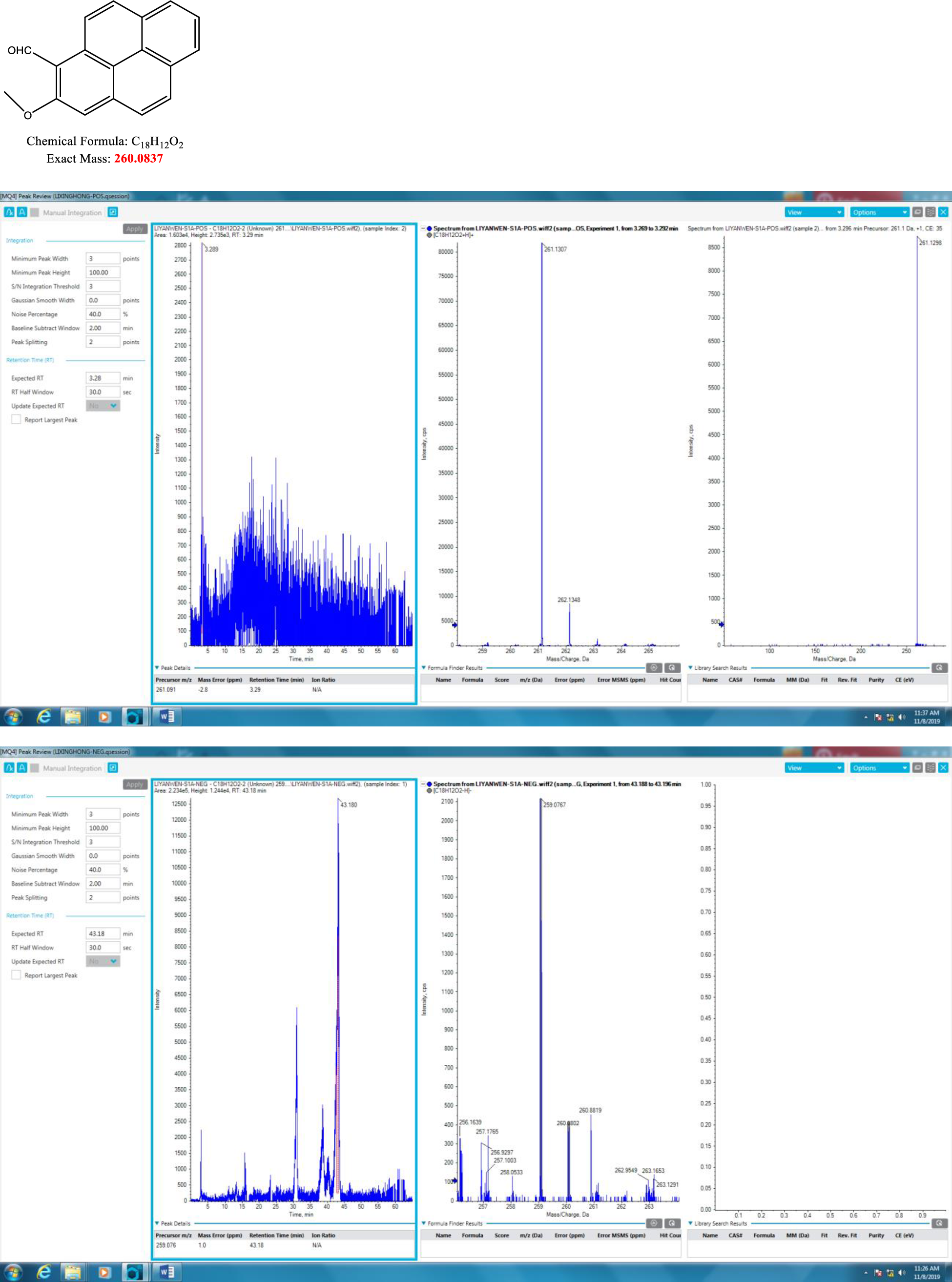

Supplement: S2 Fig — (ZIP) [file pone.0247723.s002.zip › PACE Corrected/Figure 2S Supplementary Material.tif]

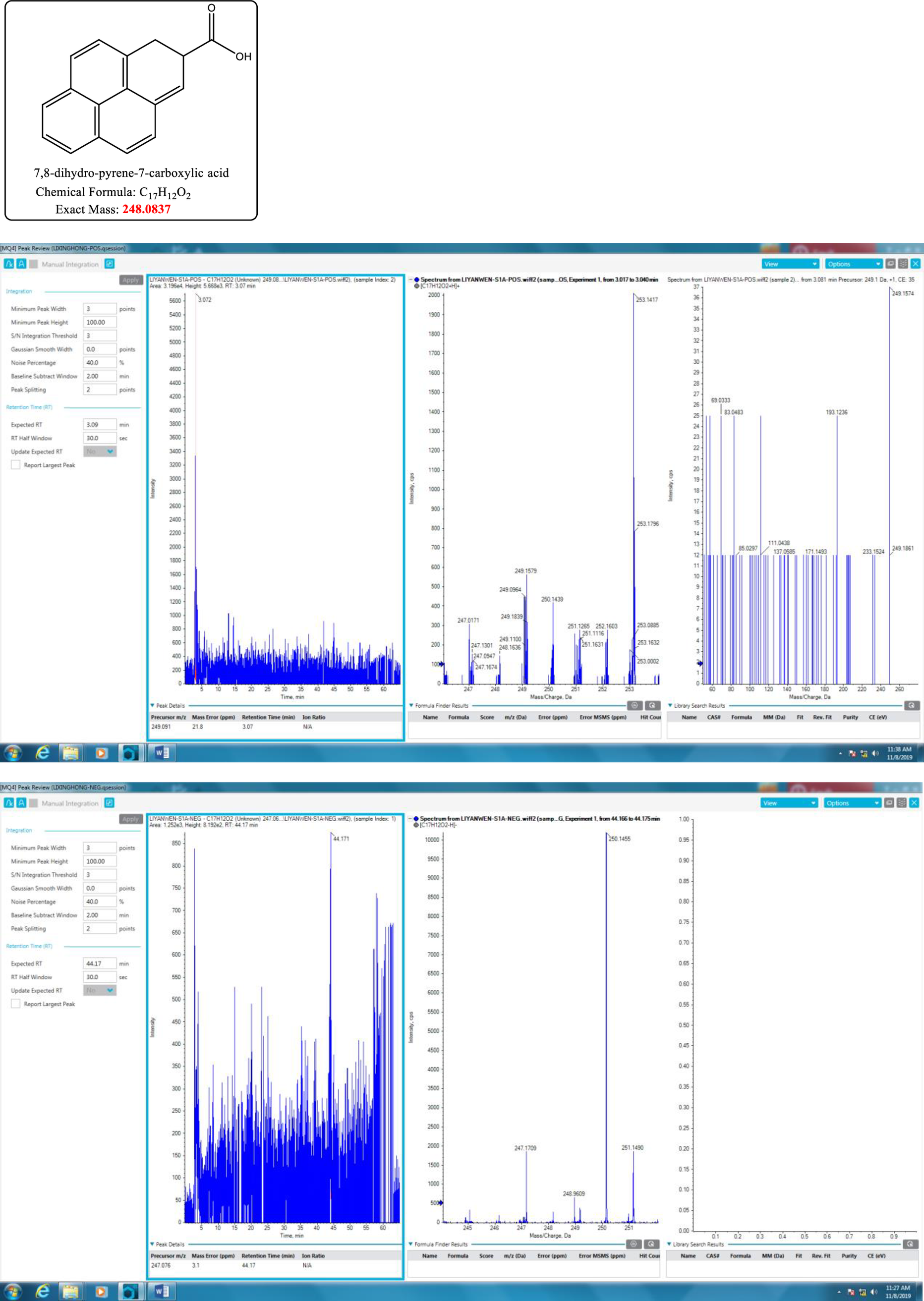

Supplement: S2 Fig — (ZIP) [file pone.0247723.s002.zip › PACE Corrected/Figure 2S Supplementary Material.tif]

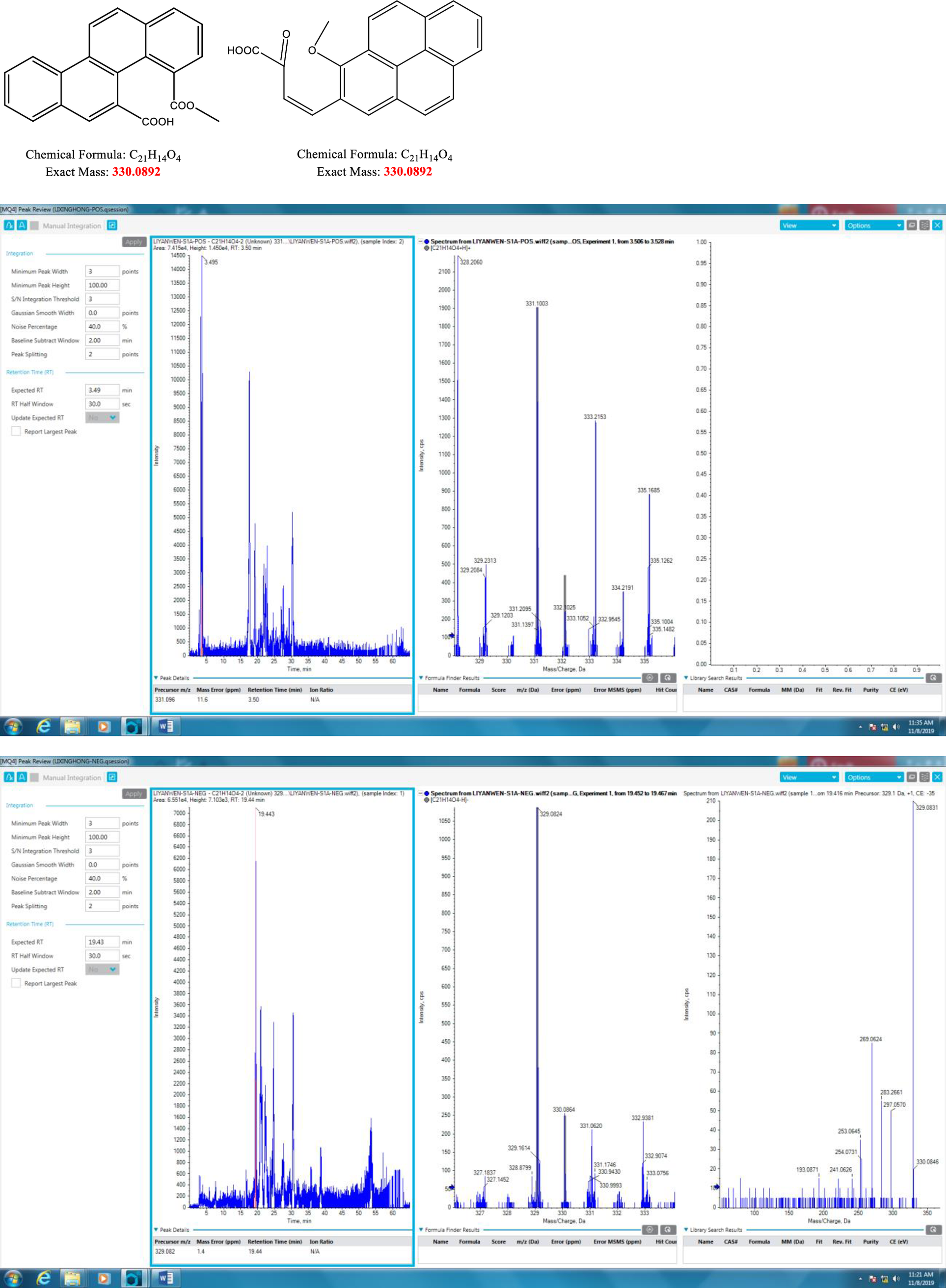

Supplement: S2 Fig — (ZIP) [file pone.0247723.s002.zip › PACE Corrected/Figure 2S Supplementary Material.tif]

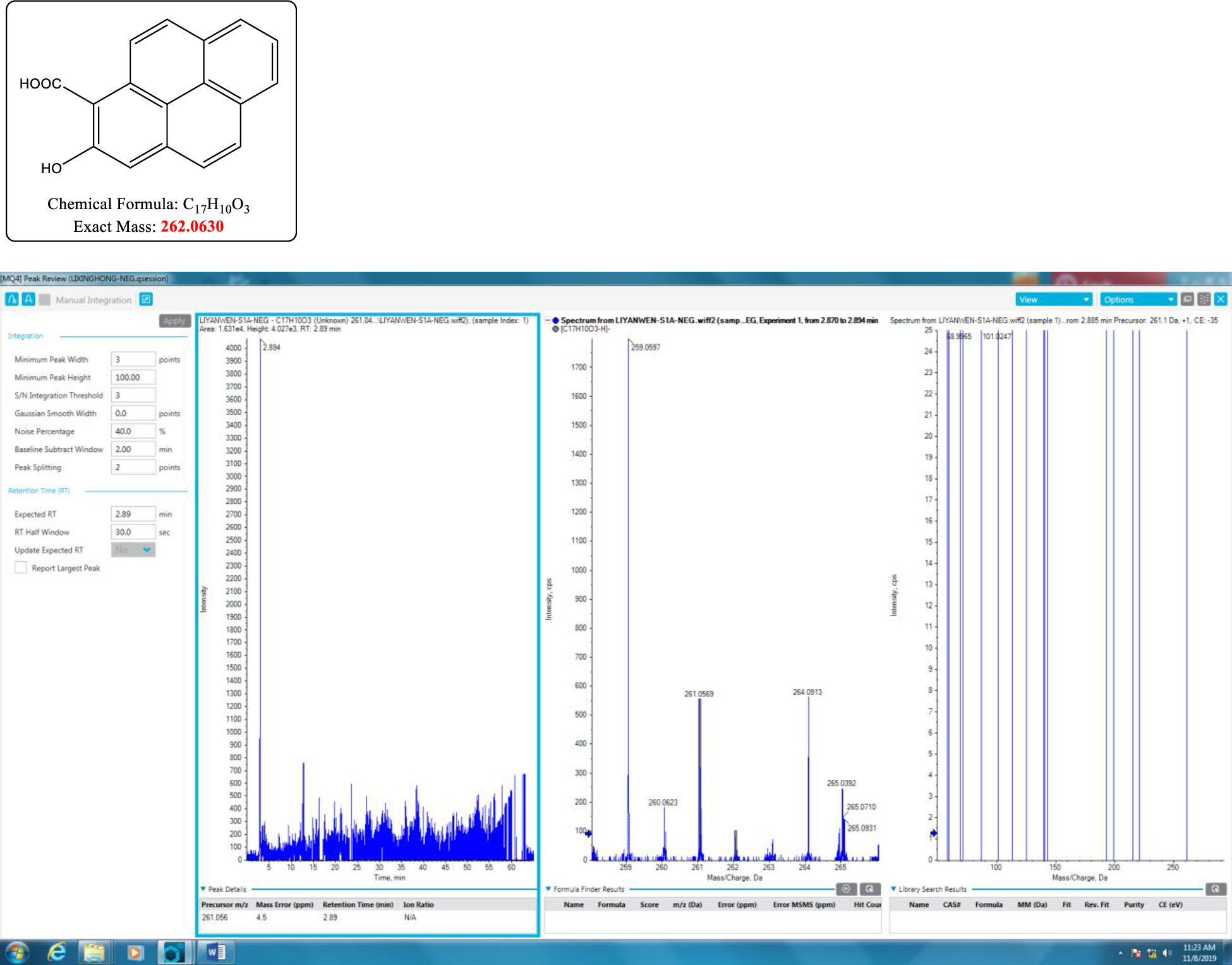

Supplement: S2 Fig — (ZIP) [file pone.0247723.s002.zip › PACE Corrected/Figure 2S Supplementary Material.tif]

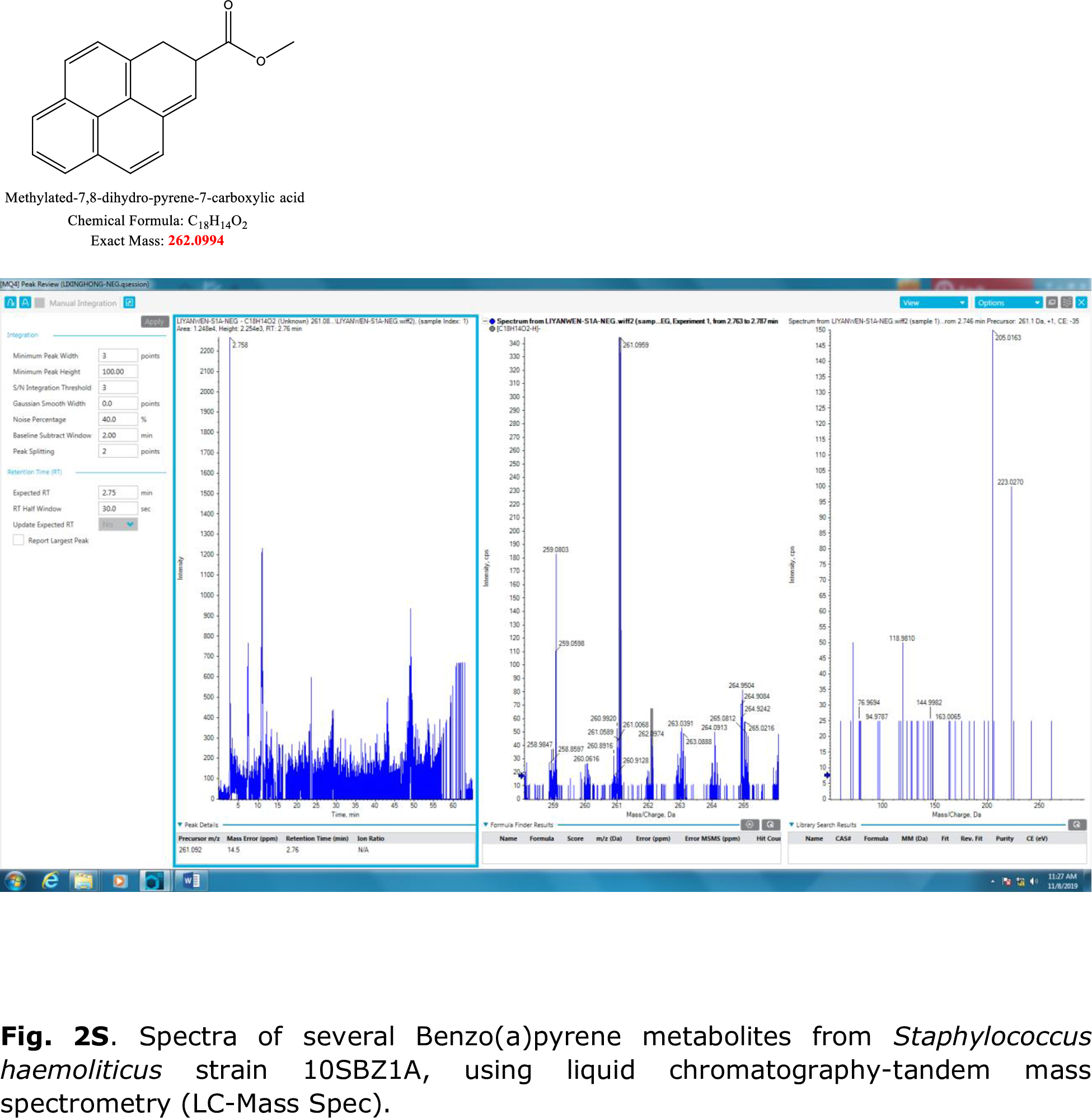

Supplement: S2 Fig — (ZIP) [file pone.0247723.s002.zip › PACE Corrected/Figure 2S Supplementary Material.tif]
