# Supplementary material for: Degradation of benzo[a]pyrene by halophilic bacterial strain Staphylococcus haemoliticus strain 10SBZ1A
Source: PLoS One. 2021 Feb 25;16(2):e0247723. doi: 10.1371/journal.pone.0247723 (PMC7939701; doi:10.1371/journal.pone.0247723)
Supplement: Attachment — Submitted filename: Response to Reviewer_PONE-D-20-38549_.docx [file pone.0247723.s004.docx]

PONE-D-20-38549
Degradation of benzo[a]pyrene by the halophilic bacterium strain Staphylococcus haemoliticus, 10SBZ1A
PLOS ONE

**POINT BY POINT RESPONSE TO THE REVIEWERS’ COMMENTS**

1. Please ensure that your manuscript meets PLOS ONE's style requirements, including those for file naming. The PLOS ONE style templates can be found at

**OUR RESPONSE**: *Our response: we have rechecked our manuscript about the style requirement*

b) If there are no restrictions, please upload the minimal anonymized data set necessary to replicate your study findings as either Supporting Information files or to a stable, public repository and provide us with the relevant URLs, DOIs, or accession numbers. For a list of acceptable repositories, please see <http://journals.plos.org/plosone/s/data-availability#loc-recommended-repositories>.

**OUR RESPONSE**: *We have uploaded an excel file containing the necessary set of data to replicate our findings.*

2. Please include a copy of Table 1 which you refer to in your text on page 10.

**OUR RESPONSE**: *This has been an oversight, the Table has been added*.

4. Have the authors made all data underlying the findings in their manuscript fully available?

**OUR RESPONSE**: *As mentioned earlier, we have uploaded an excel file containing the necessary set of data to replicate our findings.*

**Reviewer 1**

1. Abstract Line 42-43, Please carefully check the results of doubling time ‘2-10h’ and degradation rate ‘0.2 μmol/l’. It is inconsistent with the text.

**OUR RESPONSE**: *We thank the reviewer for this remark. Yes, there was inconsistency with the text and the figures. This has been changed accordingly.*

2. Abstract Line 42-44, “The strain 10SBZ1A degraded BaP at a rate of 0.2 μmol/l per day” “The optimum conditions of growth were 37 ºC, 10% NaCl (w/v) and pH 7.” It’s better to change the order of the two sentences. It should be the degradation rate under optimum conditions.

**OUR RESPONSE**: *We agree with the suggestion. Therefore we have changed the order of these 2 sentences.*

3. Materials and methods Line 202-208, What’s the salinity of the soil sample? Please add the operation conditions of this experiment, including temperature, salinity, and pH.

**Our response**: *The soil was not used dried. It was kept in the medium solution (BH), pH 7, salinity 5%. We have clarified this in the text.*

4. Introduction Line 60-66, this part introduced the background of this study. I advise the authors to supply some references of benzo[a]pyrene as the main PAHs components existed in the produced water. In addition, some references are also needed to support the high salinity of PW, even up to 30% NaCl.

**OUR RESPONSE**: *We quoted reference (Ref.1), that is relevant in relation with produced water (PW). In light of the reviewer comment, we have added an additional reference (Ref.2), and this reference provides information that PAHs are part of important pollutants found in PW.*

**Reviewer #2**: 

1. Delete the word "the" from the title and there should be "bacterial" in place of "bacterium".

***OUR RESPONSE****: Done*

2. The end of the sentence is incomplete "and among them, Benzo[a]pyrene (BaP)" at Line No. 34-35. Rewrite.

***OUR RESPONSE****: We have removed “among them” and replace it with “including”. The sentence is not clearer.*

3. Cite the following reference at the end of the sentence "The biodegradation of HMW-PAHs is challenging........difficult it is to degrade": Arora (2020), doi: 10.3389/fbioe.2020.570307

***OUR RESPONSE****: Done*

4. Delete "also" from the sentence at Line no. 35.

***OUR RESPONSE****: Done*

5. Rephrase the sentence " that can reach up to 30% NaCl" at Lie No. 35-36.

***OUR RESPONSE****: This has been done, by replacing*" that can reach up to 30% NaCl" by *“*which can be as high as 30% NaCl”

6. There should be "organic contaminants" in place of "PAHs" at Line no. 75.

***OUR RESPONSE****: In this sentence, we are referring to a specific type of the organic pollutants, which are PAHs. Thus, replacing PAHs will make the sentence not clear.. So we suggest to keep it.*

7. Mention the purity status of the followings: pyrene, anthracene, phenanthrene, naphthalene, phthalic
120 acid, salicylic acid, catechol; palmitic, oleic and stearic acids.

***OUR RESPONSE****: All chemicals were of HPLC, analytical grades. We have added this information in the text.*

8. Write the model, make and country of origin for GC at Line No. 208.

***OUR RESPONSE****: We have provided the necessary additional information of the GC equipment we have used*

9. Why the chromatograms of liquid chromatography-high resolution tandem mass spectrometry, gas chromatography-mass spectrometry and HPLC is not included in the main text. Include all these chromatogram and supporting information in the main text of the manuscript.

***Our response****: This information has been added as Supplementary material. Adding this information in the text is going to make it unnecessary long. Such information is appropriate to be added as Supplementary material.. Thus, for the conciseness of the manuscript, we suggest to keep it as Supplementary material.*

END DOCUMENT
