# Supplementary material for: Degradation of benzo[a]pyrene by halophilic bacterial strain Staphylococcus haemoliticus strain 10SBZ1A
Source: PLoS One. 2021 Feb 25;16(2):e0247723. doi: 10.1371/journal.pone.0247723 (PMC7939701; doi:10.1371/journal.pone.0247723)
Supplement: Attachment — Submitted filename: Reviewer Response.docx [file pone.0247723.s005.docx]

***Point by point response to the editor comments***

1. There are two full-stop line number 44.

***Our response:*** *This has been removed*

2. Line 33, produced water is not scientific term. Use petroleum waste effluent or something else in whole manuscript.

***Our response:*** *The term produced water is used in many publications, for instance, in Ref1 &2, the term “produced water” is even mentioned in the titles.. However, in light of the editor comments, we have mentioned that produced water is same as* petroleum waste effluent (line 34 and 61), that we have replaced “produced water” by “petroleum waste effluent (PWE)” throughout the text.

3. use word " Strain before 10SBZ1A in whole manuscript.

***Our response:*** *This has been changed throughout the text, the tables and the figures*

4. Line 258- Bacterial DNA was isolated and purified.

***Our response:*** *This has been done*
5. Check other errors.
***Our response:*** *We have cross-checked the all text for mistakes.*

*END DOCUMENT*
